# A Novel Modification of Subcutaneous Onlay Endoscopic Repair of Midline Ventral Hernias With Diastasis Recti: An Indian Experience

**DOI:** 10.7759/cureus.26004

**Published:** 2022-06-16

**Authors:** Pravin H Shinde, Vaishnavi Chakravarthy, Rajiv Karvande, Kaushik Mahadik, Jignesh Gandhi

**Affiliations:** 1 General Surgery, Seth Gordhandas Sunderdas (GS) Medical College and King Edward Memorial (KEM) Hospital, Mumbai, India., Mumbai, IND; 2 General Surgery, Seth Gordhandas Sunderdas (GS) Medical College and King Edward Memorial (KEM) Hospital, Mumbai, IND; 3 Plastic Surgery, All India Institute of Medical Sciences, Bhubaneswar, Bhubaneshwar, IND

**Keywords:** umbilical hernia, epigastric hernia, laparoscopic ventral hernia repair, scola, diastasis recti

## Abstract

Introduction

A ventral hernia is a common problem in the population. Many patients with umbilical/epigastric hernia often present with diastasis recti (DR) too. Diastasis recti is the thinning of the linea alba with an abnormal increase in the distance between the recti without a concomitant fascial defect. The presence of diastasis recti complicates the repair of the existing umbilical/epigastric hernia. Repair of only the umbilical/epigastric hernia in the presence of DR results in incomplete repair and predisposes to recurrence. There are various options available for the repair of umbilical hernia with diastasis recti. Open hernia repairs often have unsatisfactory cosmetic outcomes and, furthermore, involve complications frequently associated with large incisions such as surgical site occurrences (SSO), pain, dermal flap necrosis, and delayed postoperative recovery, to name a few. The era of minimal access surgery leaves us with a vast array of creative solutions to the same. Laparoscopic onlay repair has been given various names in literature, e.g., minimally invasive linea alba reconstruction (MILAR), pre-aponeurotic endoscopic repair (REPA), endoscopic linea alba reconstruction (ELAR), subcutaneous onlay laparoscopic approach (SCOLA), and totally endoscopic assisted linea alba reconstruction (TESLAR), with similar principles for all the procedures. The average rate of seroma formation in these procedures varies from 5% to 40%. SCOLA has been used in our study, with an added modification of the operating port and limiting the extent of lateral dissection with the aid of spinal needles, resulting in restrained dissection and creation of smaller lipocutaneous flaps, leading to reduced incidence of seroma formation.

Methods

Patients with symptomatic primary ventral hernia with concomitant diastasis recti were enrolled in the participating center from the period of May 2020 to December 2021. Thirty patients were enrolled for this prospective study. The patients underwent subcutaneous laparoscopic onlay repair of midline ventral hernia with diastasis recti, with plication of the defect and onlay placement of a polypropylene mesh.

Results

Six point sixty-six percent (6.66%) of the patients developed seroma and SSO. The incidence is congruent with the results available in current literature. None of the patients had necrosis of umbilical skin. There were no recurrences at the three months follow-up.

Conclusion

Our modification of SCOLA is an ergonomically favorable procedure and has comparable outcomes to other approaches, with minimal complications.

## Introduction

Diastasis recti (DR) is a common diagnosis complicating the repair of umbilical/epigastric hernias. Currently, the options available for the treatment of DR with umbilical/epigastric hernia are many, with varying rates of complications, e.g. seroma formation [1-4]. The prevalence of concomitant DR with umbilical/epigastric hernia is about 45% [5]. The linea alba has variable width throughout the abdominal wall, with Beer et al. describing the distance between the two recti as follows: at the level of the xiphoid process, the distance is 7 ± 5 mm (upto 15 mm), at a level 3 cms above the umbilicus, 13 ± 7 mm (upto 22 mm), and 8 ± 6 mm (upto 16 mm) at a point 2 cms below the umbilicus [6]. For all practical purposes, DR is identified when the distance between two recti exceeds 22mm. Ranney has classified DR on the basis of the above distance with W1 including DR upto 3cms, W2 with DR of 3-5cms, and W3 with DR of more than 5 cms [7]. The same has been included in the European Hernia Society (EHS) scheme of classification of ventral midline hernias. The EHS classification also takes into account the location of the midline hernia as follows: M1 (subxiphoidal), M2 (epigastric), M3 (umbilical), M4 (infraumbilical), and M5 (suprapubic) [8]. Both the umbilical/epigastric hernia and DR have to be addressed simultaneously. There are various options available. The open methods of repair have obvious drawbacks of poor cosmesis, SSO, pain, etc. We have a vast range of minimally invasive procedures available to us such as intraperitoneal onlay mesh repair (IPOM), minimally invasive linea alba reconstruction (MILAR), endoscopic linea alba reconstruction (ELAR), endoscopic pre-aponeurotic repair (EPAR), and sub-cutaneous onlay laparoscopic approach (SCOLA), to name a few. We implemented the SCOLA technique for the repair of ventral (umbilical/epigastric) hernia, plication of DR, and mesh placement, with some modifications to the traditional technique. The technique described below has outcomes comparable to other procedures, with superior ergonomics and similar rates of recurrence.

## Materials and methods

**Figure 1:**
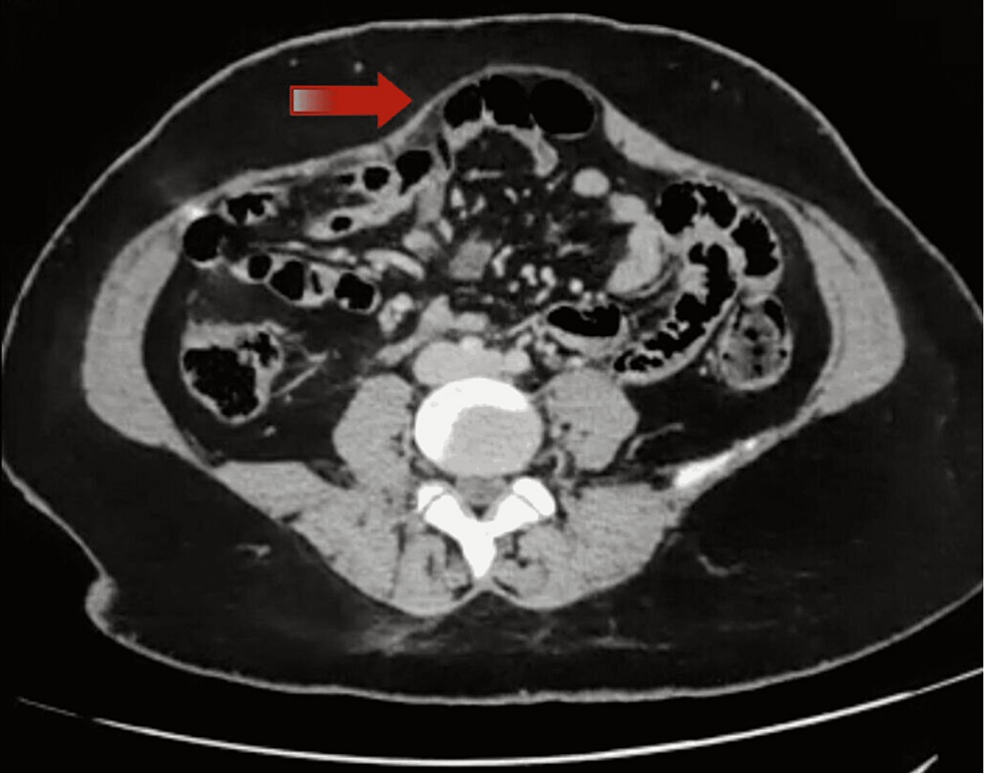
CT scan depicting divarication of recti

The study has been initiated after due approval from the institutional ethics committee of Seth Gordhandas Sunderdas (GS) Medical College and King Edward Memorial (KEM) Hospital. Patients above the age of 18 years with umbilical/epigastric hernias with concomitant DR have been included in the study. Patients with defects of more than 4 cm, incisional/port site hernia, and lack of fitness for general anesthesia have been excluded from the study. Due preoperative workup was done with preoperative computed tomography (CT) documenting the defect size. Figure 1 shows CT demonstrating diastasis recti. Data regarding the operative details were collected intraoperatively. Postoperative follow-up was done at one and three months. Details regarding recurrence were collected via patient reporting.

Surgical technique

The patient is placed in a lithotomy position. Alternatively, the procedure may also be done with the patient in a supine position with the left arm tucked in and the right arm abducted. In this scenario, the surgeon stands on the right side of the patient, and the monitor is kept on the left. This particular position can be employed in cases without divarication. The lithotomy position offers an advantage over this in patients with concomitant DR. The surgeon is located in between the legs of the patient. For the purpose of ergonomics, it is ideal if the surgeon is seated during the initial part of the surgery and is standing for the purposes of suturing and plication of the defect and DR.

**Figure 2:**
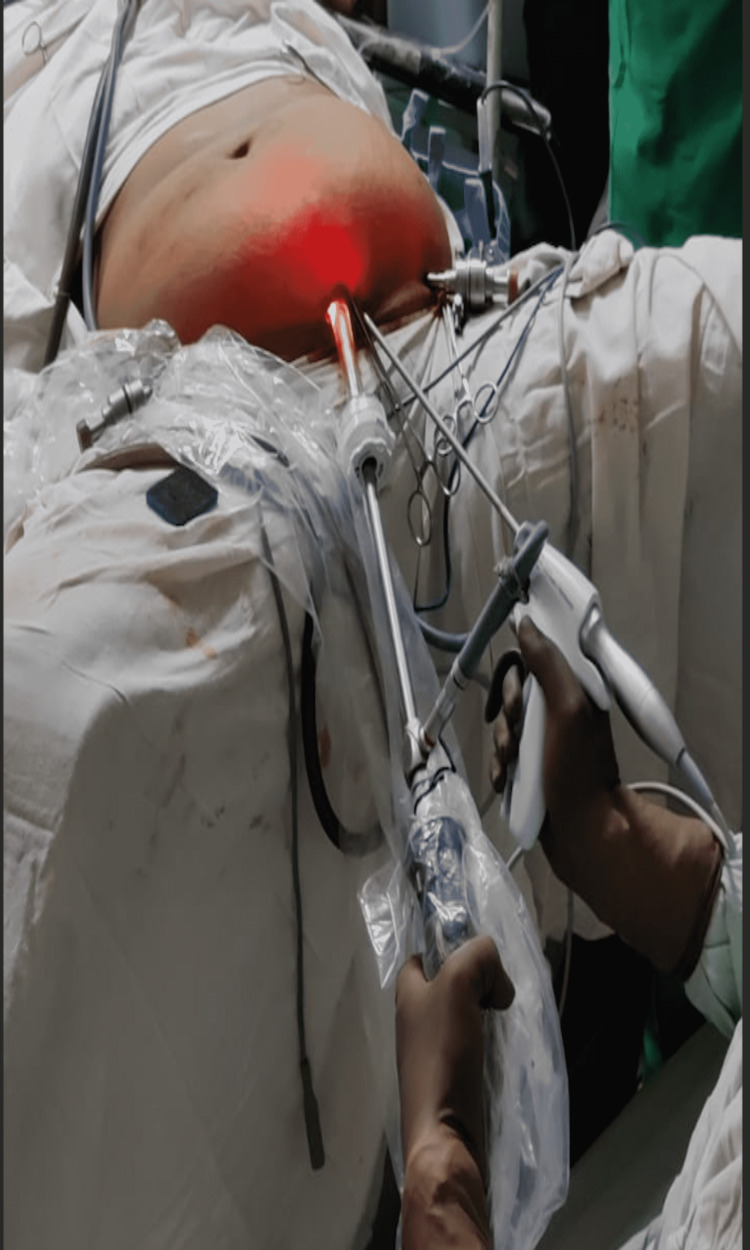
Figure depicting the position of the surgeon between the patient's legs

Figure 2 depicts the above-mentioned positioning.

**Figure 3:**
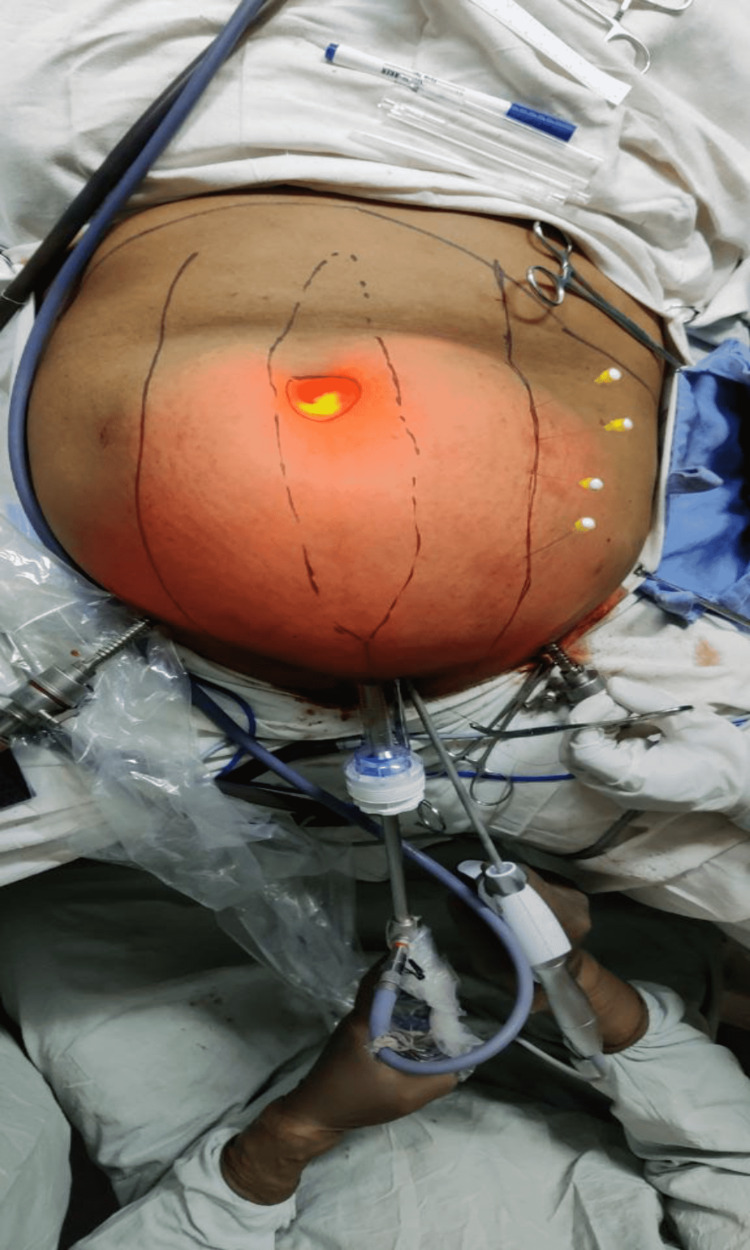
Lateral extent of dissection marked by spinal needles

The access port is obtained with a 2 cm transverse suprapubic incision, with blunt dissection done till the anterior rectus sheath (ARS) is reached. A 12 mm optical port (optiport) is inserted through this and used as a camera port. Carbon dioxide was insufflated through this port to maintain a pressure of 10-12 mmHg and the port was secured to the dermis with the help of a non-absorbable monofilament purse-string suture to prevent gas leak. We have added a modification to this access port and introduced a harmonic scalpel through the same incision alongside the optiport. This provides the surgeon with ease of operability, allowing dissection to proceed on either side of the midline. Figure 3 depicts the modification made to the suprapubic port. If the harmonic scalpel is introduced from the lateral port, creating the plane along the midline can be challenging. Two other 5 mm ports are inserted, positioned 6 cms laterally on either side of the first port, along the same line. The dissection starts towards the hernial sac on either side of the midline, creating a tunnel along the same path. The hernial sac is dissected all around and reduced. Care is taken not to use any form of energy source near the umbilical tube. The umbilical tube is divided with cold scissors to avoid any ischemia, which may happen with the use of an electrosurgical unit (ESU). The anterior layer of the rectus sheath is dissected away from Scarpa’s fascia on either side of the midline for around 5-6 cms or till the linea semilunaris is reached. The dissection also proceeds upwards approximately 5 cm superior to the location of the hernia.

The defect is marked. The extent of lateral dissection is marked with a skin marker and a spinal needle is inserted through this line. Figure 3 depicts the same. Figure 3 also depicts the modifications made to the suprapubic port.

**Figure 4:**
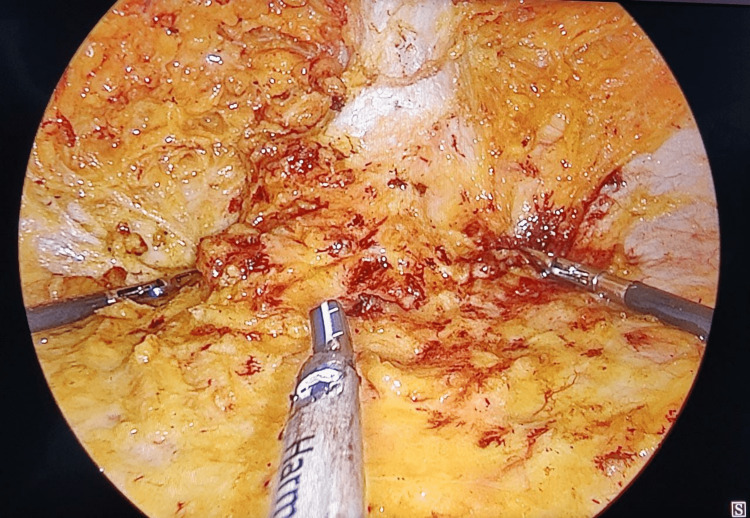
Dissection proceeding towards umbilical hernia

Figure 4 depicts the dissection upwards towards the umbilical hernia. 

**Figure 5:**
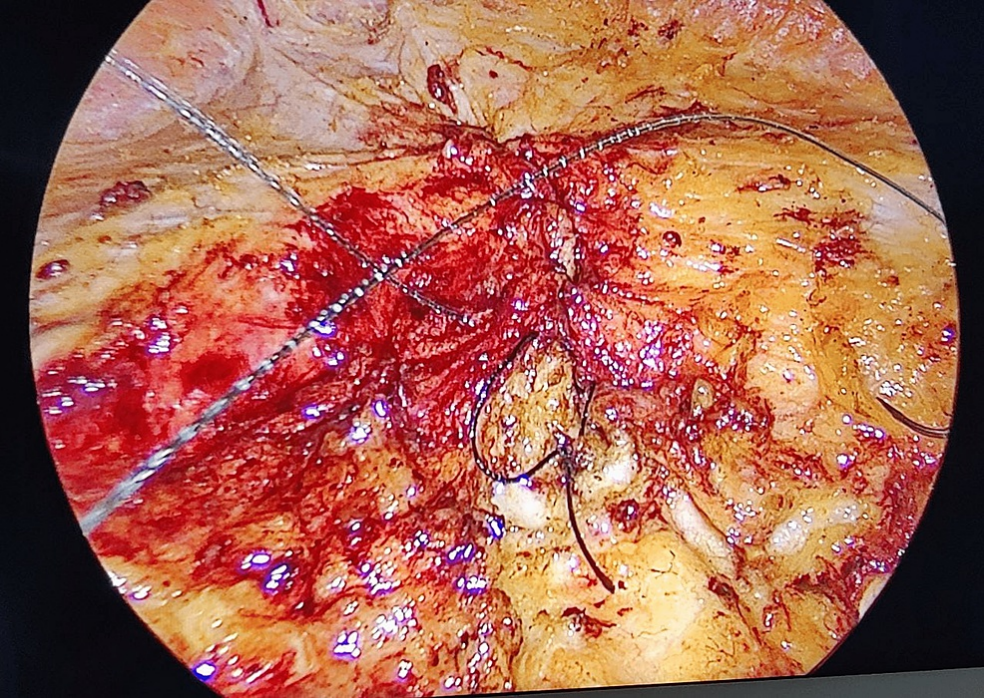
Plication of defect with barbed suture

Care should be taken to avoid excess lateral dissection. Excessive lateral dissection leads to the creation of large lipocutaneous flaps, which are associated with various complications such as seroma formation, SSO, and necrosis of the flaps due to compromised blood supply. The blood supply of the umbilicus is derived from the perforating branches of the deep inferior epigastric artery. Excessive lateral dissection can disrupt these perforating branches, thereby interfering with the viability of the umbilical skin. We have devised a method to prevent overzealous lateral dissection by marking the limits of dissection by the insertion of a spinal needle through the skin. The needles are visualized intraoperatively, thus guiding the lateral extent of dissection. The DR is visualized as a depression between the two recti and marked. The defect is suture-closed and the DR is plicated with continuous delayed absorbable barbed sutures (2-0 polydioxanone), as depicted in Figure 5. The dissected area is measured with a sterile scale. An appropriately sized, medium-weight macroporous polypropylene mesh is then inserted through the 12 mm port and fixed to the anterior rectus sheath (ARS) with tackers. Alternatively, the mesh can be fixed with interrupted sutures.

**Figure 6:**
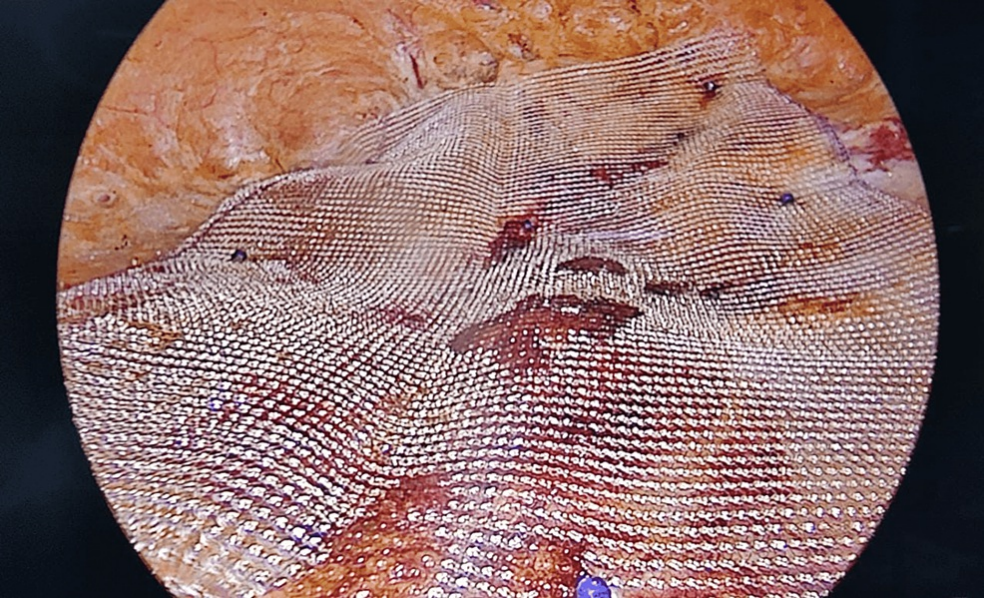
Mesh placement in the preaponeurotic plane

Figure 6 depicts the mesh, which has been fixed with tackers. Two subcutaneous Jackson-Pratt drains are inserted. The drains are generally kept till the output is below 30 ml.

## Results

A total of 30 patients fulfilling the inclusion criteria were enrolled in the study from the period of May 2020 to December 2021.

Table 1 shows relevant data collected during the study.

**Table 1:** Relevant data regarding patient characteristics and complications

Parameter	Value
Age in years (Mean)	42.30
Males	20 (66.66%)
Females	10(33.33%)
Mean BMI	28.9
Defect size (Mean)	2.10
Primary hernia	30
Mesh Type - Polypropylene	30
Mesh fixation with tackers	20
Mesh fixation with interrupted sutures	10
Surgical site infection	2
Seroma	2
Flap necrosis	0

The mean BMI of this study population was 28.9 kg/m^2^. The mean defect size was 2.10 cm. Six point sixty-seven percent (6.67%) of the patients developed surgical site infection (SSI) and seroma. Flap necrosis and recurrence were not observed. Patients fulfilling the inclusion criteria were enrolled in the study. The average operating time was approximately 110±10 minutes. There were no significant intraoperative complications. None of the procedures was converted to open surgery. Two patients developed seroma postoperatively. It was managed conservatively with close observation for signs of infection and resolved spontaneously within a month. Jackson-Pratt drains were removed after the output reduced to less than 30 ml per day. Two patients developed superficial SSI and were treated conservatively with antibiotics and daily dressings. No early recurrences were seen.

## Discussion

**Figure 7:**
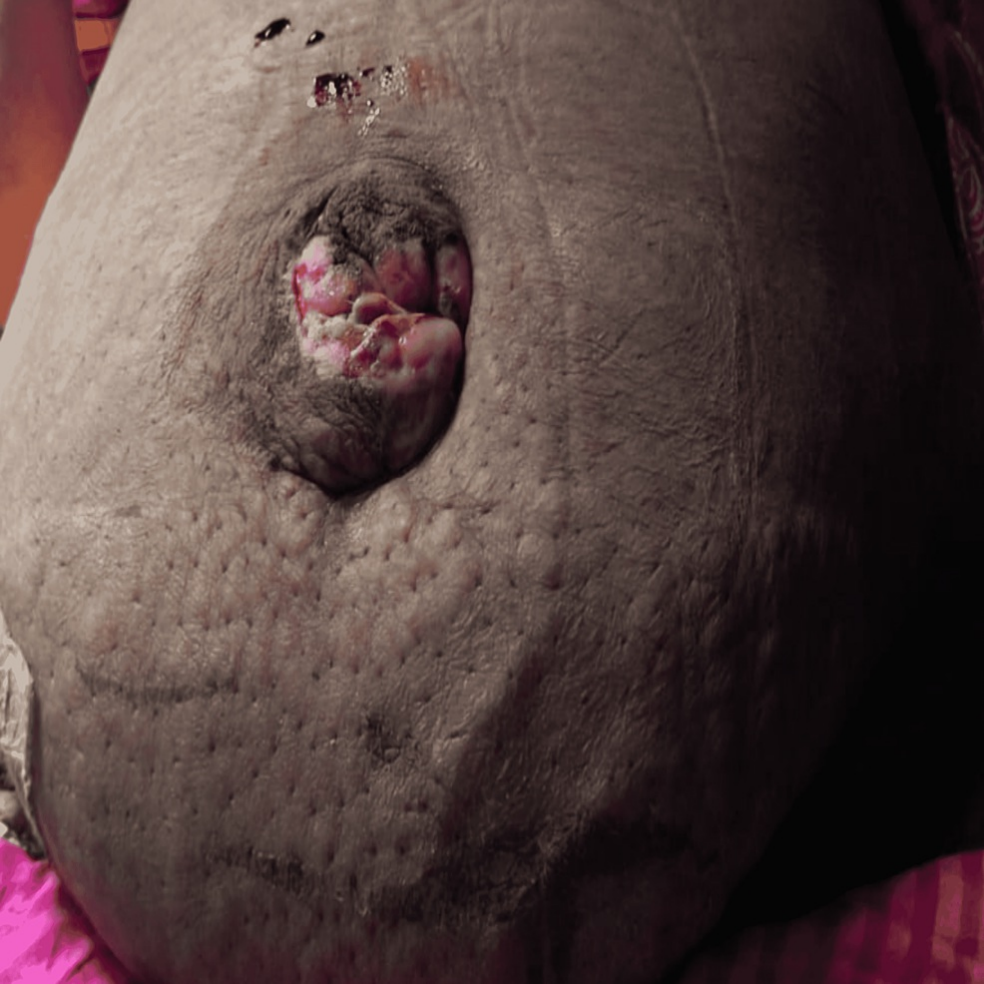
Umbilical necrosis due to excessive dissection in the subcutaneous plane

An umbilical hernia is a common surgical problem that is quite often accompanied by diastasis recti. Factors predisposing to DR are central obesity, ascites, smoking, multiparity, and decreased core muscle strength. DR itself adds to the core muscle instability and may result in spinal deformities, such as hyperlordosis, and constipation along with an undesirable lump in the abdomen. These lumps may only become clinically visible on head raising or leg raising tests. Quite understandably, these are a source of mental and physical discomfort to the patient. The abdominal wall reconstruction armamentarium has a variety of techniques for treating the same. Each technique has its advantages and disadvantages, and ultimately, the choice of surgery is based on the available technical expertise, patient factors, and preference of the patient. Over the last few decades, minimally invasive techniques have been extensively refined. Minimal access surgery offers clear advantages over open surgery. The postoperative recovery is faster, the severity of pain is lesser, and patients have better cosmetic results. We have several options for the repair of diastasis recti with concomitant umbilical/epigastric hernia. Champault was the first to describe the videoendoscopic repair of DR, and it was indicated in patients with well-preserved skin over an abdominal wall defect [9]. There are various other techniques for the repair of DR, many of them similar, with notable modifications. Kockerling and Kohler described the ELAR and MILAR procedures. MILAR is distinguished by the lack of videoendoscopic equipment [10]. A small incision is made over the abdomen and with the help of lighted retractors, dissection is carried out in the pre-fascial plane. Myofascial release of the rectus with concomitant plication of the defect and placement of an absorbable mesh is carried out. Claus et al. and Dong et al. have described their SCOLA experience in Brazil and the US, respectively [11]. We present our experience of this procedure for ventral hernia with DR at a tertiary care setup in India. We have added a novel modification to the technique. Insertion of a spinal needle 5-6 cm from the midline on either side helps guide the lateral dissection. The blood supply of the abdominal wall is via the superior and inferior deep epigastric artery. The musculocutaneous perforators provide the cutaneous blood supply and these are concentrated around 5-6 cm lateral to the umbilicus. Excessive lateral dissection can result in seroma formation and may also result in the disruption of the blood supply of the abdominal wall, hence predisposing to SSI and flap necrosis [12]. Studies of similar procedures available in literature by Claus et al., Muas et al., and Kockerling et al. have shown a 5-40% recurrence rate. Kler et al. observed seroma formation in 80% of their patients, which can be attributed to the use of a larger use of biologic mesh [13-14]. Figure 7 depicts umbilical skin necrosis in which the above modification of SCOLA was not followed.

## Conclusions

Our experience shows that SCOLA is an efficient procedure for the treatment of umbilical/epigastric hernia with diastasis recti with minimal complications and postoperative morbidity. The technique gives an acceptable cosmetic result to patients. The rate of seroma formation varies widely, from 5% to 80% as mentioned above. Our added modification serves to help with the same and prevents excessive dissection leading to large lipocutaneous flaps.

Since this is a prospective study with short-term follow-up, the limitations of the study include inadequate long-term follow-up for recurrence. We would also like to perform this study on a larger sample size, so the results can be extrapolated to the general population.
